# *Mycobacterium bovis* Pulmonary Tuberculosis, Algeria

**DOI:** 10.3201/eid2703.191823

**Published:** 2021-03

**Authors:** Fatah Tazerart, Jamal Saad, Abdellatif Niar, Naima Sahraoui, Michel Drancourt

**Affiliations:** Université Ibn Khaldoun de Tiaret, Tiaret, Algeria (F. Tazerart);; Université de Blida, Blida, Algeria (F. Tazerart, N. Sahraoui);; Institut Hospitalo-Universitaire Méditerranée Infection, Marseille, France (F. Tazerart, J. Saad, M. Drancourt);; Aix-Marseille-University, Marseille (J. Saad, M. Drancourt);; Laboratoire de Reproduction des Animaux de la Ferme, Université Ibn Khaldoun de Tiaret, Tiaret (A. Niar)

**Keywords:** tuberculosis and other mycobacteria, *Mycobacterium tuberculosis*, *Mycobacterium bovis*, whole-genome sequencing, Algeria, bacteria

## Abstract

We analyzed 98 *Mycobacterium tuberculosis* complex isolates collected in 2 regions of Algeria in 2015–2018 from 93 cases of pulmonary tuberculosis. We identified 93/98 isolates as *M. tuberculosis* lineage 4 and 1 isolate as *M*. *tuberculosis* lineage 2 (Beijing). We confirmed 4 isolates as *M. bovis* by whole-genome sequencing.

In Algeria, interpreting tuberculosis (TB) incidence, estimated at 53–88 cases/100,000 population in 2017 (*1*), is limited by the fact that the diagnosis relies on microscopic examination of clinical samples. Isolates are presumptively identified as *Mycobacterium tuberculosis* complex based on colony phenotype.

We analyzed 98 sputum isolates identified as *M. tuberculosis* complex by 5 Tuberculosis and Respiratory Disease Control Service facilities in 2015–2018 (Appendix Table 1, Figure, ). Exact tandem repeat D analysis (*2*) confirmed these 98 isolates as *M. tuberculosis* complex. Large-sequence polymorphism analysis using PCR sequencing of genomic regions RD105, RD239, and RD750 and of the polyketide synthase gene *pks*15/1 (*3*) yielded 88 (89.8%) *M. tuberculosis* sensu stricto Euro-American lineage 4 isolates and 1 East Asian lineage 2 (Beijing) isolate. Whole-genome sequencing (WGS) of 5 RD deletion–free unidentified isolates indicated that these 5 isolates, P9982(ERR3588223), P9983(ERR3588225), P9985(ERR3588243), P9984(ERR3588246), and P9986(ERR3588247), were *M. tuberculosis* sensu stricto Euro-American lineage 4. We conducted WGS analysis using TB-profiler for *M. tuberculosis* online tool (https://tbdr.lshtm.ac.uk/upload) for lineage and sublineage determination. Altogether, *M. tuberculosis* lineage 4 was the predominant lineage in the 5 Algerian departments and the sole lineage documented in Bgayet, Tizi-Ouzou, and Medea (Appendix Table 2); it was found to be the cause of pulmonary TB in 79/93 (85%) cases, pleural TB in 11 (12%) cases, and lymph node TB in 3 (3%) cases. These observations updated those issued from a previous study conducted in 14 departments including 114 (88%) cases of pulmonary localization and 15 cases (12%) of extrapulmonary localization (*4*). In a later study, spoligotyping revealed that most isolates belonged to *M. tuberculosis* Euro-American lineage 4; the Haarlem clade accounted for 29.5% of studied isolates; the Latin American-Mediterranean clade, 25.6%; and the T clade, 24.8% (*4*). In our study, 1 *M. tuberculosis* Beijing strain was isolated from a bronchial fluid sample collected in Blida from the location at which 15 *M. tuberculosis* Beijing isolates had been identified ≈10 years earlier from 14 workers from Algeria and 1 from China (*5*). Our observation suggests that 10-year circulation of *M. tuberculosis* Beijing strain in the community in Blida area most probably followed immigration of workers from China employed in the construction sector.

WGS analysis of 4 additional isolates exhibiting a 6-bp deletion in the *pks*15/1 gene identified them as *M. bovis*. Using a Roary pangenome pipeline (https://sanger-pathogens.github.io/Roary), we found that *M. bovis* CSURP9981 grouped with *M. bovis* CSURP9979 and that *M. bovis* CSURP9980 grouped with *M. bovis* CSURP9978 (Figure). Further analysis based on the 3,732,808-bp core genome detected 3,761-bp (0.1%) of single-nucleotide polymorphisms (SNPs) between the 4 isolate genomes. Whole-genome sequences of *M. bovis* strains in the study have been deposited in GenBank (sequence P9978, accession no. ERR3587501; P9979, no. ERR3587591; P9980, no. ERR3587597; and P9981, no. ERR3588222).

**Figure:**
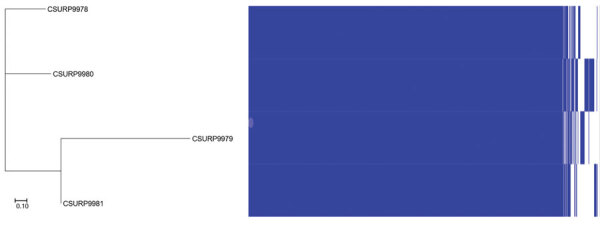
Pangenome-based tree of 4 human *Mycobacterium bovis* isolates, Algeria. The tree was generated by Roary (https://sanger-pathogens.github.io/Roary) from binary gene presence or absence in the accessory genome. Scale bar indicates 10% sequence divergence.

All 4 patients had pulmonary TB and had no detectable lymph node swelling and no scrofula (*6*). Two case-patients in Blida were a 27-year-old unemployed man and a 60-year-old taxi driver who both declared that they did not consume raw milk and had no contacts with cattle; a neighbor of the 60-year-old patient was a butcher with whom he spent a lot of time. Two case-patients in Ain Defla were 18-year-old and 43-year-old housewives living in 2 different rural areas. The interviews of these patients did not reveal contacts with cattle. Identification of human cases of *M. bovis* was unexpected because in 50 years, only 7 cases of *M. bovis* human infection have been reported in Algeria: 3 cases of pulmonary TB and 2 cases of cervical lymphatic TB detected in a total of 1,183 (0.4%) phenotypically identified *M. bovis* isolates (*7*), and 2 additional cases reported in 2009 (*8*).

*M. bovis* TB is clinically, pathologically, and radiologically indistinguishable from *M. tuberculosis*; diagnosis requires accurate identification of the causative mycobacterium, most efficiently by using WGS. Our report illustrates pitfalls in precisely tracing the natural history of *M. bovis* TB in patients, including sources, routes of transmission, and primary route of entry, which may determine the pathology of the infection. Zoonotic *M. bovis* TB was most often transmitted to humans by the consumption of *M. bovis*–contaminated dairy products that caused lymphatic TB, eventually becoming pulmonary TB (*9*). We previously reported a hidden circumstance for contacts with *M. bovis*–infected animals, tracing 1 *M. bovis* pulmonary TB case in a patient in Tunisia to contacts with an infected sheep during religious festivities in 2018 (*10*). In the case we report here, foodborne transmission cannot be ruled out, but it is possible that this may be a rare case of aerosol transmission.

Algeria is a bovine TB–enzootic country. We recommend comparing the genome sequences from the 4 patients reported here with those of future bovine isolates in the same departments to trace zoonotic *M. bovis* TB in Algeria and contribute to the understanding of its natural history.

Appendix Additional information about *Mycobacterium bovis* pulmonary tuberculosis in Algeria.
