# Supplementary material for: Mycobacterium bovis Pulmonary Tuberculosis, Algeria
Source: Emerg Infect Dis. 2021 Mar;27(3):972–4. doi: 10.3201/eid2703.191823 (PMC7920681; doi:10.3201/eid2703.191823)
Supplement: Appendix — Additional information about Mycobacterium bovis pulmonary tuberculosis in Algeria. [file 19-1823-Techapp-s1.pdf]

Article DOI: https://doi.org/10.3201/eid2703.191823

Mycobacterium bovis Pulmonary Tuberculosis, Algeria

Appendix

Appendix Table 1. Demographic characteristics of tuberculosis case-patients, Algeria, 2008–2015

	No. in each age group (%)							Total
Sex	15–24 y, n = 11	25–34 y, n = 14	35–44 y, n = 17	45–54 y, n = 14*	55–64 y, n = 10*	≥65, n = 18	Unknown, n = 14	N = 98 (100%)
M	6 (54.54 ± 15.74)	6 (42.85 ± 13.72)	10 (58.82 ± 12.30)	9 (64.28 ± 13.29)	7 (70 ± 15.27)	10 (55.55 ± 12.05)	8 (57.14 ± 13.72)	56 (57.14 ± 5.03)
F	5 (45.45 ± 15.74)	8 (57.14 ± 13.72)	7 (41.17 ± 12.30)	5 (35.71 ± 13.29)	3 (30 ± 15.27)	8 (44.44 ± 12.05)	6 (42.85 ± 13.72)	42 (42.85 ± 5.03)
	p = 0.56	p = 0.29	p = 0.15	p = 0.03	p = 0.02	p = 0.35	p = 0.29	p = 0.004

*These 2 age groups were combined (45–64 y, p = 0.001).

Appendix Table 2. Distributions of different lineages of *M. tuberculosis* complex strains in five departments in northern Algeria.

		No. strains in lineage (%)				
Department	No. strains	Lineage 1	Lineage 2	Lineage 3	Lineage 4	Lineage BOV
Bgayet	20	0 (0)	0 (0)	0 (0)	20 (100)	0 (0)
Tizi-Ouzou	06	0 (0)	0 (0)	0 (0)	06 (100)	0 (0)
Blida	40	0 (0)	1 (2.5)	0 (0)	37 (92.5)	2 (5)
Médéa	16	0 (0)	0 (0)	0 (0)	16 (100)	0 (0)
Ain Defla	16	0 (0)	0 (0)	0 (0)	14 (87.5)	2 (12.5)
Total	98	0 (0)	1 (1.02)	0 (0)	93 (94.9)	4 (4.08)

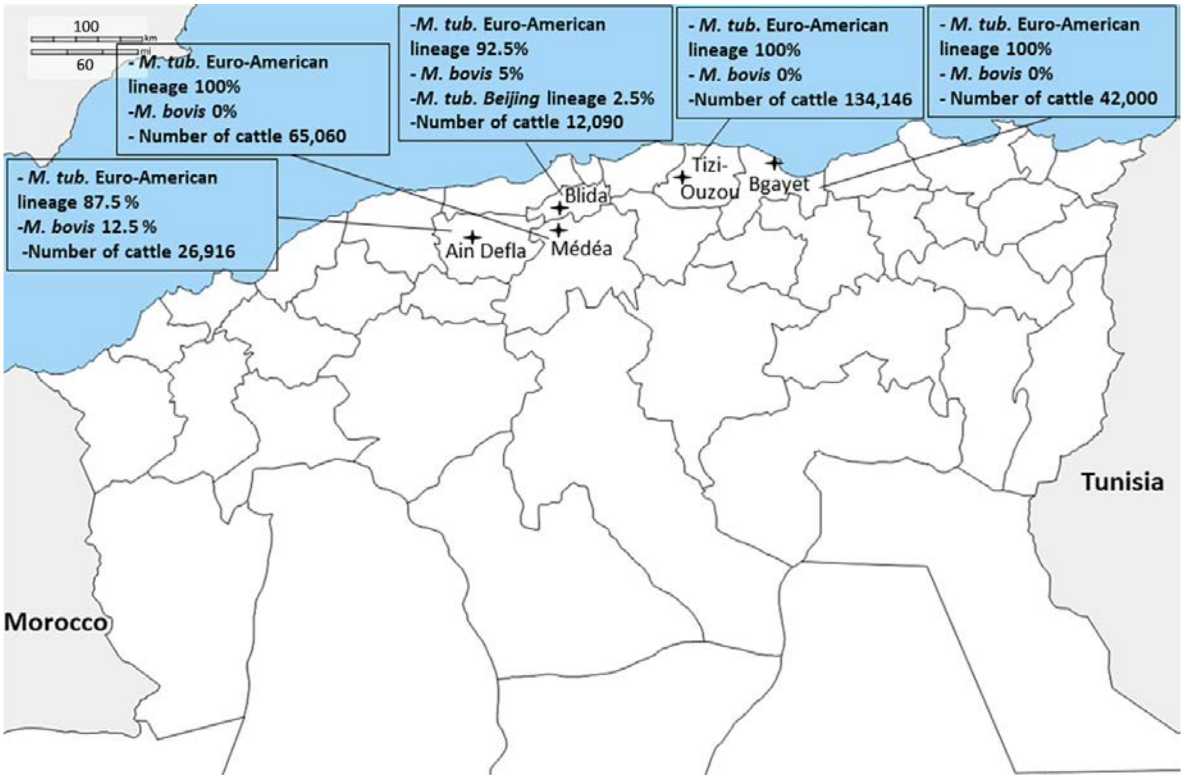

Appendix Figure. Locations of the 5 Tuberculosis and Respiratory Disease Control Service facilities located in 5 departments in northern Algeria, where *M. tuberculosis* complex isolates were collected for molecular studies. Collection period was 2015–2018 in Bgayet, 2016 in Tizi-Ouzou, 2016–2018 in Médéa, 2018 in Ain Defla, and 2018 in Blida.
